# A Bimolecular Multicellular Complementation System for the Detection of Syncytium Formation: A New Methodology for the Identification of Nipah Virus Entry Inhibitors

**DOI:** 10.3390/v11030229

**Published:** 2019-03-07

**Authors:** María J. García-Murria, Neus Expósito-Domínguez, Gerard Duart, Ismael Mingarro, Luis Martinez-Gil

**Affiliations:** Department of Biochemistry and Molecular Biology, ERI BioTecMed, University of Valencia, 46100 Valencia, Spain; Murria@uv.es (M.J.G.-M.); laneus121@gmail.com (N.E.-D.); gerard.Duart@uv.es (G.D.); Ismael.Mingarro@uv.es (I.M.)

**Keywords:** Virus entry, Bimolecular complementation, membrane fusion, Nipah virus, High-throughput screening

## Abstract

Fusion of viral and cellular membranes is a key step during the viral life cycle. Enveloped viruses trigger this process by means of specialized viral proteins expressed on their surface, the so-called viral fusion proteins. There are multiple assays to analyze the viral entry including those that focus on the cell-cell fusion induced by some viral proteins. These methods often rely on the identification of multinucleated cells (syncytium) as a result of cell membrane fusions. In this manuscript, we describe a novel methodology for the study of cell-cell fusion. Our approach, named Bimolecular Multicellular Complementation (BiMuC), provides an adjustable platform to qualitatively and quantitatively investigate the formation of a syncytium. Furthermore, we demonstrated that our procedure meets the requirements of a drug discovery approach and performed a proof of concept small molecule high-throughput screening to identify compounds that could block the entry of the emerging Nipah virus.

## 1. Introduction

Regardless of the entry pathway at some point during their life cycle, viruses must cross the cell membrane. For enveloped viruses, this process requires the fusion of the viral and cellular membranes [1]. Enveloped viruses trigger this membrane merge process by means of specialized viral proteins expressed on their surface, the so-called viral fusion proteins. By undergoing intense structural rearrangements, viral fusion proteins are capable of lowering the kinetic barriers necessary to achieve the coalition of two biological membranes. Currently three classes of fusion proteins have been characterized according to their structure and mechanism of action: Class I (e.g., influenza HA), Class II (represented by flavivirus envelope protein E) and Class III (illustrated by rhabdovirus glycoprotein G). For a detailed review on the characteristics of each class of viral fusion proteins visit [1,2]. Fusion can be triggered directly by interactions of the fusion or a companion protein at the viral surface with a cellular receptor on the host plasma membrane. In this case, the expression of the viral fusion protein together with the attachment protein at the host cell membrane during viral replication can lead to syncytia formation (fusion of neighboring cells generating multi-nucleate cells). Alternatively, the interaction between a viral and cellular protein at the cell surface can prompt particle endocytosis. Subsequently, the low endosomal pH or the interaction with a second internal receptor elicits the fusogenic conformational change required for membrane fusion [2].

Although the vast majority of antivirals focus on blocking viral replication, the key role of fusion and attachment proteins during the virus life cycle makes them an attractive target for therapeutic intervention. There are several successful entry inhibitors in the market (including Human immunodeficiency virus (HIV) and Influenza A virus (IAV) antivirals) and many more in research and development stages [3,4,5,6,7,8]. There are some advantages of targeting an extracellular protein such as the viral fusion and attachment proteins or the cellular receptors expressed on the cell surface necessary for viral entry. These types of extracellular target sites are much easier to reach for the antiviral, resulting in improved pharmaco-kynetics and lower toxicity profiles of the drug of choice. It is also advisable to have more of these types of drugs simply to increase the potential therapy combinations, a highly successful treatment regimen extensively used to fight severe infections such those produce by HIV or Hepatitis C virus [9,10].

There are multiple assays both in vitro and in vivo for evaluating viral entry, including cell-virus fusion assays with pseudotyped viral particles, cell-cell fusion assays, and in vitro biochemical assays [11]. Among these, the cell-cell fusion assays, based on the formation of a syncytium due to the expression of viral proteins on the cellular surface, offer a safe and virus free alternative. Identification of syncytium has traditionally been done by microscopy. The microscope-based methodologies are far from ideal, they are slow, not quantifiable, and lack sensitivity. Furthermore, despite huge advances in image analysis [12,13], the implementation of these methods for high throughput screenings (HTS) is not yet optimal.

Many efforts have been made to facilitate the analysis of cell-cell fusions induced by viral proteins, especially in the HIV field [11,14,15,16,17,18,19]. Herschhorn et al. [20] described a system based on the fusion of two cell lines, an effector line stably expressing a tetracycline-controlled transactivator (tTA) that controls the expression of HIV-1 Env and a target cell line expressing the HIV-1 receptors CD4 and CCR5 and the Renilla luciferase under a tTA-responsive promoter. Env-mediated fusion of these two cell lines grants tTA dependent activation of the F-Luc expression. In an earlier manuscript [21], Bradley J. and colleagues reported a similar methodology using two cell lines, one expressing CD4, CCR5, and the β-galactosidase under a LTR-promoter, while the other expresses constitutively HIV proteins gp160 and tat. Fusion of these two cell lines facilitates the transfer of the tat transcription factor and the accumulation of β-galactosidase. Interestingly, the authors probed that this methodology could be used in a HTS compatible format. Even the use of split reporters has been explored to investigate cell-cell fusion events triggered by viral proteins [22]. These techniques have proven useful for the development of anti-HIV drugs. However, they are not compatible with other viruses and require the generation of cell lines, which despite having some advantages (mainly the standardization of the results) is time consuming and hinders the analysis of mutants and variants of the proteins involved in the process.

Nipah virus (NiV), an emerging zoonotic virus from the Paramyxoviridae family, includes in its membrane: the viral encoded fusion (F) and attachment (G) proteins. Attachment to the target cell is achieved by the G protein binding to the Ephrin B2 receptor on the cell surface [23,24]. This interaction activates the F protein and in turn, the fusion of the viral and cellular membranes. Therefore, the expression of the NiV F and G proteins at the host cell membrane are sufficient to induce syncytia formation. Currently, there is no antiviral or approved vaccine for this deadly virus, and subsequently it has been classified as a Biosafety Level 4 (BSL4) pathogen. In this manuscript, we describe a novel methodology for the study of cell-cell fusion, utilizing NiV F and G proteins as the model system. Our approach, named Bimolecular Multicellular Complementation (BiMuC), is based on the bimolecular complementation of a reporter [25] and provides a platform to safely investigate the entry process of viruses, such as NiV, which encode fusion proteins that induce the formation of cellular syncytia when expressed on the cellular surface. The adjustability of our methodology facilitates the investigation with other viruses as long as a cell-cell fusion event can be triggered. Furthermore, we demonstrated that our procedure meets the requirements of a drug discovery approach and performed a proof of concept small molecule HTS to identify compounds that could block the entry of the emerging NiV.

## 2. Materials and Methods

### 2.1. Plasmids and Cell Lines

HEK 293T cells were obtained from ATCC (http://www.atcc.org) and were maintained in Dulbecco’s Modified Eagle Medium (DMEM) (Gibco, http://www.lifetechnologies.com) supplemented with 10% fetal bovine serum (FBS, Gibco).

The NiV F and G plasmids were a gift from Dr. M. Shaw laboratory (Ichan School of Medicine at Mount Sinai). These plasmids include the full-length F (Uniprot ID: Q9IH63) or G (Uniprot ID: Q9IH62) sequence in a pCAGGS background. The Jun-Nt VFP and Fos-Ct VFP expressing plasmids were obtained from Addgene (#22012 and #22013 respectively). Finally, the Jun-Nt luciferase and Fos-Ct luciferase plasmids were obtained by substituting the VFP sequence on the Jun and Fos BiFC constructs by the appropriated luciferase segment obtained from the Promega (Nt 1-229, Ct 230-311) (pRL_CMV plasmid) [26].

### 2.2. Cell-Cell Fusion Assay

For the cell-cell fusion assay, HEK 293T cells were seeded in 6 well plates (2 × 10^6^ cells/plate) on day 1 (DMEM supplemented with 10% FBS). After 24 hours (day 2), cells were transfected, using polyethylenimine (PEI) as a transfection reagent [27], with Jun-Nt, Fos-Ct, NiV F and G or mock transfected (1 µg of DNA/well). Alternatively, for the 2-pool cell approach one set of cells was transfected with Jun-Nt, NiV F and G while the other received just Fos-Ct. On day 3, cells were counted, mixed into the positive (Jun-Nt, Fos-Ct and NiV F and G expressing cells) and negative controls (Jun-Nt, Fos-Ct and mock transfected cells) and seeded into 24 (1 × 10^5^ cells/well in 500 µL of media), 96 (3 × 10^4^ cells/well in 100 µL of media) or 384 well plates (1 × 10^4^ in in 25 µL of media). In those experiments, in which only two cell pools are used, the positive control included cells expressing Jun-Nt, NiV F and G and Fos-Ct, while the negative control contained only mock-transfected cells. Finally, on day 4, fluorescence was measured on 96 or 384 well plates as previously described [28]. Alternatively, when using the 24 well plates, cells were collected and transferred into a 96 black plate prior to fluorescence measurement. Each experiment was performed at least three times in triplicates. The same protocol was used when the fluorescence reporter was substituted by a luminescence readout. In this case, we utilize 96 well white cell-culture plates and the Renilla Luciferase Assay kit from Sigma following the manufacturer protocol.

### 2.3. Identification of NiV cell-cell Fusion Inhibitors

For the identification of NiV F and G inhibitors, the aforementioned protocol was adjusted to meet the HTS criteria of the Screening facility at the Centro de Investigación Principe Felipe (CIPF) (Valencia, Spain). To evaluate the robustness of our assay we calculated the Z′-factor [29] and the Signal-to-Noise (S/N) ratio. Z′-factor = 1 − ((3δ_pos_ + 3δ_neg_)/(µ_pos_ − µ_neg_)), where µ_pos_ is the mean signal for the positive control, µ_neg_ is the mean signal for the negative control, δ_pos_ is the standard deviation of the positive control, and δneg is the standard deviation for the negative control. S/N = (µ_pos_ − µ_neg_)/((δ_pos_) × 2 + (δ_neg_) × 2) × 0.5.

Briefly, HEK 293T cells were seeded in 15 cm dishes (5 × 10^6^ cells/dish) using DMEM supplemented with 10% FBS. After 24 h (day 2) of incubation (37 °C, 5% CO_2_), cells were transfected using PEI with Jun-Nt, Fos-Ct, NiV F and G (3 µg of per plasmid) or mock transfected, as described previously. On day 3, cells were counted, mixed into the positive and assay samples (Jun-Nt, Fos-Ct and NiV F and G expressing cells) and the negative controls (Jun-Nt, Fos-Ct and mock transfected cells) and seeded into 96 well plates (3.5 × 10^4^ cells/well in 100 µL of media). In each mixture, equal amounts of cells transfected with the different plasmids were included. Next, 2 µL of a 50 µM compound stock (in 5% DMSO) was delivered into the cells (final concentration of 0.1% DMSO) using a MultiChannel Arm MCA 96 (Tecan, Männedorf, Switzerland). We used a Prestwick Chemical library (Sigma, St. Louis, MO, USA) containing 1120 compounds (note: currently the commercially available library contains 1280 compounds). In each plate, 8 wells were reserved as a positive control (treated with DMSO) and 8 wells as a negative control (did not contain NiV F and G expressing cells). After 48 h of incubation, fluorescence was measured directly on the plates in an EnSight multiplate reader (Perking Elmer, Waltham, MA, USA). Results obtained from the screen were standardized using the Z-Score, calculated as follows: Z-Score = (x − μ)/δ, where x is the raw signal, μ is the mean signal and δ is the standard deviation of all the compound-containing wells of one plate. The Z-Score indicates how many standard deviations a particular compound is above or below the mean of the plate. Primary hits were identified by calculating a Z-score for each compound and applying hit selection criteria; Z-Score < 2.

To corroborate the anti-fusion activity, selected compounds from the previous round were reanalyzed using the BiMuC methodology, once again in a 96 well format. Additionally, we measured the toxicity of the drugs by determining the number of viable cells utilizing the CellTiter 96 AQueous Non-Radioactive Cell Proliferation Assay (Promega, Madison, WI, USA) following the manufacturer instructions. Only those compounds with a Z-Score < 2 in both rounds of the fusion assay and with a cell viability above 90 % (compared to the vehicle treated samples) were selected as hits.

## 3. Results

### 3.1. Assay Design

**Figure 1:**
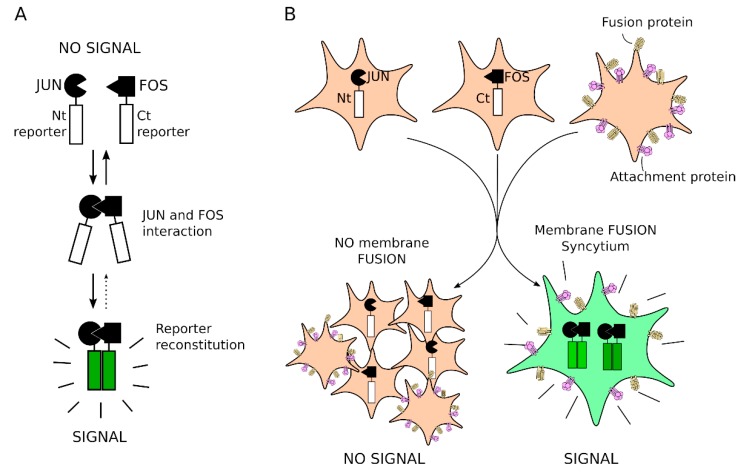
Schematic representation of Bimolecular Multicellular Complementation (BiMuC) assay. (**A**) Representation of the Bimolecular Complementation chimeras. (**B**) Schematic representation of the BiMuC assay.

We aimed to design a methodology for the study of cell-cell fusion triggered by viral proteins. For this purpose, we exploited the bimolecular complementation properties of the Venus Fluorescent Protein (VFP) [25,28,30,31]. The reconstitution of a split VFP, a technique known as BiFC (Bimolecular Fluorescence Complementation) assay, has been extensively used for more than a decade for the identification and investigation of protein-protein interactions, even in genome wide analysis [32]. Briefly, the VFP can be divided into two non-fluorescent fragments (amino terminal (Nt) and carboxy terminal (Ct)). Reconstitution of the VFP native structure, and thus fluorescence, from these two fragments can be achieved if the Nt and Ct halves are brought into close proximity. This approximation might be prompted by the fusion of the VFP Nt and Ct fragments to two proteins or domains that spontaneously interact, e.g., Jun and Fos [33] (Figure 1A).

To analyze the cell-cell fusion, the Nt and Ct VFP segments fused to their corresponding interaction partners are expressed in two independent cell pools, while the viral proteins required the formation of the syncytium are transfected into a third pool of cells (Figure 1B). After mixing these three cell populations, a successful fusion of the cell membranes by the viral proteins will bring the Nt and Ct moieties together and the fluorescence of the VFP will be reconstituted. Moreover, if the syncytium is not formed, no signal will be retrieved since the VFP fragments remain in different cells (Figure 1B). This methodology enables a straightforward study of cell-cell fusion processes. Furthermore, it could be applied for the identification of inhibitors of viral triggered membrane fusion and thus identification of antiviral agents.

### 3.2. Assay Validation

**Figure 2:**
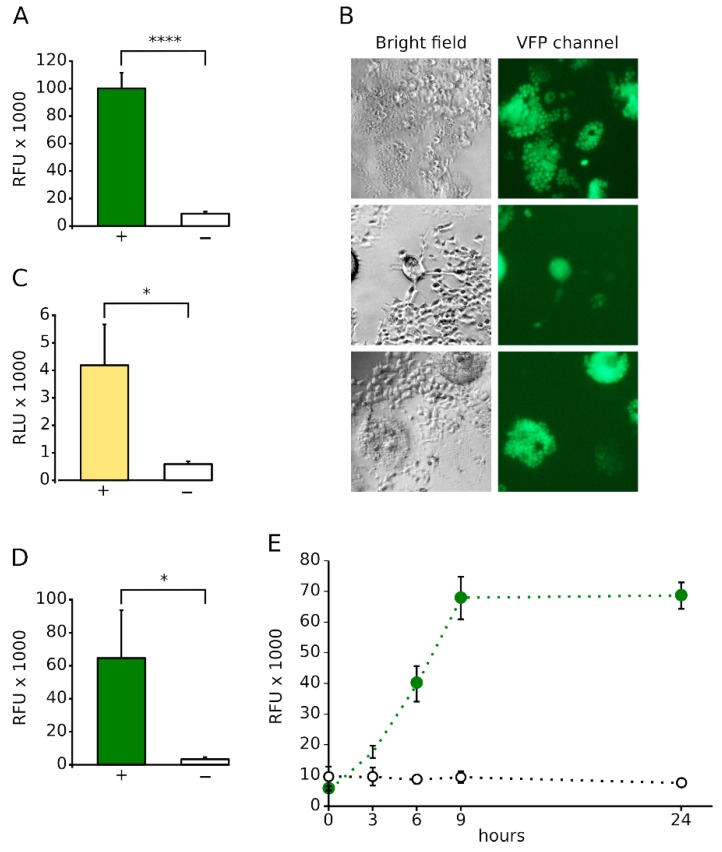
BiMuC assay validation. (**A**) Bar graph representing the fluorescence measurements results of positive (+, green including the NiV G and F, the Jun-Nt Venus Fluorescent Protein (VFP) and the Fos-Ct VFP cell pools) and negative (−, white, Jun-Nt VFP, Fos-Ct VFP, and mock transfected cells) controls. RFU, Relative Fluorescence Units. (**B**) Bright and VFP channel fluorescence micrographies of positive samples in which the VFP is used as a reporter. As expected, only fused cells can reconstitute the VFP signal. Three micrographies including syncitia of different sizes and at multiple stages have been included. All three pictures were taken at the same magnification. (**C**) Bar graph showing the relative luminescence units (RLU) of the positive (+) and negative (−) for the BiMuC assay in which the luciferase has been used as a reporter. * *p*-value < 0.05, **** *p*-value < 0.0001. (**D**) Results of the 2 cell-pools approach using VFP as a reporter. Once again, the positive control (+) is depicted with a green bar while the negative control (−) is shown using a white bar. (**E**) Kinetics of the fusion process. RFU at multiple times (hours) post cell pool combination for the positive (green) and negative (white) controls. Error bars denote standard deviation of at least three replicates.

To test our hypothesis, we independently transfected the Jun-Nt VFP and Fos-Ct VFP chimeras into HEK 293T cells [25,33,34,35,36]. Alongside, a third pool of cells was transfected with the NiV F and G proteins (Figure 1B), which have been reported to be sufficient for cell-cell fusion [37,38] or mock transfected. After transfection, cells were mixed into two groups, both containing Jun-Nt and Fos-Ct expressing cells. Additionally, the positive group (+) was supplemented with cells expressing NiV F and G proteins while in the negative control (−) mock-transfected cells were included. After 24 h, the fluorescence of both groups was measured. The results showed a staggering difference between samples with or without the viral proteins (Figure 2A). Only those samples that received the viral proteins (+), and thus formed a syncytium, were able to reconstitute the VFP. As expected, a fluorescence microscopy analysis of samples including the split VFP as a reporter, revealed that cells that undergo cell-cell fusion are fluorescent while single cells remain non-fluorescent (Figure 2B). Figure 2B shows three images (taken at the same magnification) including syncytia of multiple sizes and at several times post transfection. These results confirm the potential of our methodology for the analysis of cell-cell fusion events through the complementation of the VFP moiety.

Many enzymes have shown the same reconstitution properties as the VFP [39]. For this reason, we decided to test whether the proposed methodology could be used with the luciferase (a widely used light producing enzyme), as a syncytium formation reporter. Similarly to the formerly described approach, we fused Jun and Fos to the Nt and Ct ends of the Renilla luciferase [40]. Next, we repeated the experiment using the three-cell pool as previously described but using the new luciferase-based split reporter. The results (Figure 2C) demonstrated that the luciferase is tolerated as a reporter by the system, which further increase our confidence in the devised methodology. We believe that the flexibility of the assay regarding the reporter increases the potential number of applications for our method.

Alternatively, we designed a protocol in which only 2 cell pools are required for the assay. In this case, one set of cells were transfected with Jun-Nt, NiV F and G proteins while the other received just Fos-Ct. Once the cells were mixed and the syncytium was formed a strong fluorescent signal was observed (Figure 2D). Contrarily, mock transfected cells remained non-fluorescent. This methodological variation represents a simplified, but equally efficient version of the assay. However, due to the increased variability observed using this last described method, we decided to employ for subsequent analysis the original design in which three pools of cells are mixed.

Next, we decided to explore the kinetics of the fusion process. For this experiment, we proceeded using the three-pool approach, and the VFP as reported. After mixing the three cell pools, the samples were incubated for 3, 6, and 9 h and the results compared with the 24 h-incubation formerly described (Figure 2E). Two main conclusions can be obtained with this assay. First, the fusion process (at the present conditions) reaches its maximum after nine hours. Second, the methodology allows a kinetic analysis of this type of fusion process since the signal increases steadily throughout the assay until the 9-h mark.

### 3.3. A Bimolecular Multicellular Complementation System for High-Throughput Small Molecule Identification

**Figure 3:**
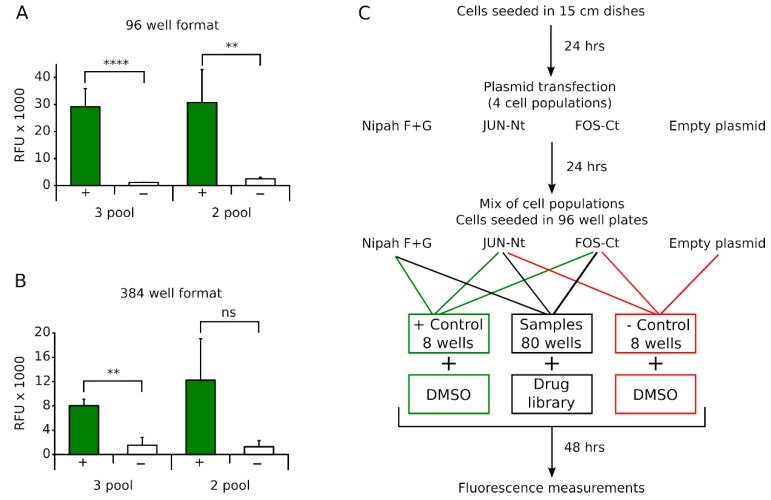
Miniaturization of the assay. BiMuC assay using the VFP as a reporter in a 96 (**A**) and 384 (**B**) well format; 3 pool and 2 pool indicates whether the 3 cell pools or the 2 cell pools approach was used. Positive control (+), negative control (−). Error bars denote standard deviation of at least three replicates. ** *p*-value < 0.01, **** *p*-value < 0.0001. (**C**) Schematic representation of the BiMuC assay high-throughput workflow.

The sensitivity, simplicity and customization possibilities of the proposed methodology are ideal for the identification of new viral fusion and attachment proteins inhibitors. These small molecule screenings are performed in a high (or medium)-throughput compatible format. Consequently, we decided to analyze the performance of our assay in 96 and 384 wells plate formats. The results (Figure 3), indicate that our assay is suitable for a 96 and even a 384 well format, regardless of whether we use 2 or 3 sets of cells.

Prompted by these results, we decided to perform a small molecule screening as a proof of concept. The assay was performed in a 96 well format at the Screening laboratory of the Centro de Investigación Principe Felipe (Valencia, Spain). We verified the suitability of our protocol for use in a High-throughput screening (HTS) by the Z′ factor (Z′ = 0.77) [29] and the signal-to-noise ratio (S/N = 12.99). For the screening, HEK 293T cells were seeded in complete media and 24 h later transfected with Jun-Nt, Fos-Ct, NiV F and G or mock transfected (Figure 3). After transfection, cell pools were mixed and seeded as described above and each well treated with the appropriated small molecule. In each plate, 8 wells were reserved as a positive control (treated with DMSO) and 8 wells as a negative control (did not contain NiV F and G expressing cells). Next, cells were incubated 48 h allowing the small molecule to act, followed by fluorescence measurement and hit selection on a decreased signal over the vehicle-treated cells.

A library consisting of 1120 chemically diverse small molecules, most of them approved drugs, (Prestwick Chemical) was screened and the results standardized by the Z-Score (primary hits were selected based on a Z-Score of <2). We identified 16 hits, which represent a hit rate of 1.4%. After a hit confirmation, using the same experimental conditions, and a cell proliferation assay where those compounds with a toxicity above 10% were eliminated the number of hits was reduced to 7 (hit rate = 0.62%). Selected hits are included in Table 1.

Several of the identified compounds were previously reported as entry inhibitors (Table 1) for Herpes simplex virus (HSV) [43] or Hepatitis B virus (HBV) [45]. Interestingly, these compounds are currently used as cardiac stimulants [46]. Those small molecules unidentified as viral inhibitors are described in the literature, as protein synthesis inhibitors (see Table 1 for references).

## 4. Discussion

Viral infections have an enormous impact on global health. Just in the USA, it is estimated that influenza infections alone are responsible for 610,660 deaths and 3.1 million hospitalized days with an associated economic burden of $87.1 billion [47]. Thus, the prevention and treatment of viral infection must be a priority. In general, the treatment of viral infections often relies on anti-viral small molecules. Direct-acting antivirals (DTA) (antivirals that target the virus life cycle) are tremendously efficacious on blocking the viral replication [48]. Unfortunately, we have a very limited range of DTA at our disposal. Furthermore, some of the current blockbusters on viral inhibition might not remain so due to apparition of viral resistance [49]. Therefore, we must keep working on the development of new and improved antivirals. However, the discovery of DTA has proven challenging at best, particularly, for BSL3 and BSL4 pathogens due to the restrictions imposed by the bio-safety regulations. To facilitate the development of new entry inhibitors, we have designed a methodology that allows the quantification of cell-cell fusion events in a simple manner.

Our procedure, based on the bimolecular complementation properties of protein reporters such as the VFP or the Renilla luciferase, facilitates the identification and quantification of cell-cell fusion events without the assistance of a microscope-based technologies. Our results clearly indicate that this approach is robust and reproducible (*p*-value < 0.0001 was observed between the positive and negative samples, Figure 2A). Furthermore, our assay displays a great flexibility; we could substitute the Venus fluorescent protein by the Renilla luciferase as a reporter or simplify the assay by using two cell pools instead of three and were still able to obtain solid results (Figure 2C,D). We used HEK 293T cells because they support NiV replication and can be easily transfected. Nonetheless, the assay, as described in this manuscript, can be performed in any other cell type as long as significant transfection efficiency is achieved. In theory, we could apply our approach, not only to the study of viral induced cell-cell fusion but also to any other cellular process, in which a syncytium is formed, e.g., the formation of the muscle fibers. The system is based on syncytium formation and thus it necessitates the surface expression of the viral proteins inducing the cell-to-cell membrane fusion. Therefore, the assay works within a physiological pH range and would not be applicable to analyze the low-pH triggered membrane fusion induced by some viruses.

We also utilized the described methodology for the identification of small molecules capable of inhibiting the syncytium formation driven by the expression of NiV F and G proteins. In our screening, we have identified several steroidal glycosides as NiV inhibitors. Interestingly, these compounds were previously described as HSV and HBV inhibitors as well [43]. Nonetheless, this class of drugs, inhibitors of Na + /K + -ATPase, were thought to act at early stages of HSV replication and at the virus release stage but not, as our data indicate, as virus entry or attachment inhibitors [43]. All the accumulated data regarding steroidal glycosides indicate that membrane rearrangements requires an intact concentration of ions across the cellular membrane. Therefore, several stages of the viral life cycle that require membrane rearrangements might be blocked by steroidal glycosides.

In conclusion, we have developed a new, simple, versatile, and safe methodology for the identification and quantification of syncytium formation applicable in small scale for an in-depths analysis of the cell-cell fusion process and in large scale to HTS small molecule identification of viral inhibitors.

## Figures and Tables

**Table 1:** Inhibitors of NiV F and G induced cell-cell fusion.

Name	Formula	MW	Reported as Inhibitor of
Anisomycin	C14H19NO4	265.31	Protein synthesis [41]
Cephaeline dihydrochloride heptahydrate	C28H54Cl2N2O11	665.66	Protein synthesis [42]
Digitoxigenin	C23H34O4	374.53	HSV [43]
Digoxin	C41H64O14	780.96	HSV [43]
Strophantine octahydrate	C29H60O20	728.79	HSV [43]
Emetine dihydrochloride	C29H42Cl2N2O4	553.58	Protein synthesis [44]
Proscillaridin A	C30H42O8	530.66	HBV [45]
